# Editorial: Non-Coding RNAs: Entwining Metabolism and Aging

**DOI:** 10.3389/fendo.2018.00111

**Published:** 2018-03-19

**Authors:** Marcelo A. Mori

**Affiliations:** ^1^Department of Biochemistry and Tissue Biology, Institute of Biology, University of Campinas, Campinas, Brazil

**Keywords:** microRNAs, long non-coding RNA, aging, senescence, cardiovascular diseases, adipose tissue, type 2 diabetes, lysosomal storage diseases

**Editorial on the Research Topic**

**Non-Coding RNAs: Entwining Metabolism and Aging**

Non-coding RNAs (ncRNAs) have emerged as important regulatory molecules in a wide range of biological processes. Among these processes, ncRNAs are notorious for their role in determining cell identity, differentiation, and metabolism. The past decade has seen a growing body of evidence that has implicated ncRNAs in multiple human diseases, in particular chronic, age-related degenerative diseases including type 2 diabetes, neurodegeneration, cancer, and cardiovascular dysfunction. ncRNAs have been linked to the pathophysiology of these diseases in part due to their impact on metabolic pathways, although these mechanisms still remain elusive, given the broad diversity of ncRNA species and their heterogeneous, largely uncharacterized and rather complex mode of action and regulation. ncRNAs have also been shown to act as signaling molecules as some species are secreted into the circulation and act in distant sites to regulate gene expression (1, 2). These functions are even less characterized, as it remains to be found how these ncRNAs are released into blood stream and which conditions regulate this process. microRNAs are perhaps the best characterized species of ncRNAs with regulatory functions and are differentially expressed in response to aging or dietary interventions that impact metabolism (3–5). microRNAs have also been implicated in type 2 diabetes and other age-related metabolic diseases (6, 7). The same has been observed, although less extensively, for other ncRNAs (8–10).

In this Research Topic, we discuss and provide new evidence for the importance of ncRNAs in metabolism and their contributions to aging and age-related diseases.

A new frontier in biology is understanding the complexity and biological importance of different forms of ncRNAs. Ziegler and Kretz focus on long non-coding RNAs (lncRNAs) and provide examples of their diversity and broad modes of action. They also discuss strategies and drawbacks to study these lncRNAs, alerting for the complexity of such enterprise.

Within the narrow universe of functionally annotated lncRNAs, some stand out as important regulators of cellular senescence. These lncRNAs are reviewed by Degirmenci and Lei. The authors also acknowledge that the field of lncRNA biology is in its infancy but given the growing body of evidence of their association with physiological processes, many more lncRNAs are expected to be linked to age-related dysfunction in the future, including in humans.

The other papers in this Research Topic focus on microRNAs and their roles in age-related, cardiometabolic diseases. Of interest to their authors is the overlap between obesity and aging. Both conditions are strongly associated with the appearance of highly prevalent non-communicable diseases (data from the World Health Organization). Shamsi et al. provide a detailed description of microRNAs with roles in adipose tissue, paying particular attention to microRNAs controlling the thermogenic potential of brown and beige adipocytes. This is important due to the established function of adipose tissue microRNAs in determining adipocyte identity (7, 11, 12) and controlling organismal aging and age-associated insulin resistance (13). On the other hand, in an original research article, Frias Fde et al. demonstrate an association between skeletal muscle microRNAs (usually referred to as “myomiRs”) and the onset of insulin resistance in mice exposed to high fat diet. Correlations between the expression levels of myomiRs and the insulin-growth factor 1 and mTOR pathways are also established. Interestingly, these pathways have been causally linked to aging in model organisms and humans (14).

Finally, two articles propose new microRNA disease associations in the context of aging. Queiroz et al. discuss the role of microRNAs in lysosomal storage diseases—a group of human genetic disorders where lysosomal proteins are deficient. The clinical manifestations of such disorders are diverse, and their causes are well defined, but many of them have been linked at some level to microRNA dysregulation. In their perspective, Queiroz et al. start from the standpoint that lysosomes play important roles in cellular physiology and metabolism, including in processes such as autophagy and vesicle trafficking, and in diseases such as neurodegeneration, and propose that one can use lysosomal storage diseases to assess the mechanisms through which lysosomal dysfunction impacts on human physiology, asking whether ncRNAs participate.

Sfera et al. close the Research Topic by hypothesizing that miR-29 is a biomarker of cerebrovascular adverse effects in elderly people exposed to antipsychotic drugs. They come up with this hypothesis by identifying a seemingly unrelated association between antipsychotic drugs, small vessel disease, and miR-29 target genes. They concluded that miR-29 upregulation may lead to a major vulnerability for cardiovascular complications, paving the way for studies that wish to test this hypothesis experimentally.

In conclusion, this Research Topic brings insights into new areas of investigation involving ncRNAs, aging, and metabolism, thereby setting the stage for new discoveries in the field.

## Author Contributions

The author confirms being the sole contributor of this work and approved it for publication.

## Conflict of Interest Statement

The author declares that the research was conducted in the absence of any commercial or financial relationships that could be construed as a potential conflict of interest.
